# ICCVAM: Not Doing Enough

**DOI:** 10.1289/ehp.1001969

**Published:** 2010-05

**Authors:** Jessica Sandler

**Affiliations:** Regulatory Testing Division, People for the Ethical Treatment of Animals, Norfolk, Virginia, Email: JessicaS@peta.org

Anyone interested in the facts about the Interagency Coordinating Committee on the Validation of Alternative Methods (ICCVAM) and its ineffectiveness, rather than just another ICCVAM/National Toxicology Program (NTP) fluff piece (Birnbaum and Stokes 2010), should read the 2008 front page *Washington Post* exposé of ICCVAM (Gaul 2008) and the PETA report on which the *Post* investigation was based (PETA 2008).

Birnbaum and Stokes’ “PR piece” notwithstanding, ICCVAM should be held responsible for failing to abide by its Congressional mandate to support the development and implementation of non-animal testing methods.

Sadly, it appears that the new leadership of the National Institute of Environmental Health Sciences is no more inclined to improve the quality of the science supporting regulatory decision-making than the previous one.
